# Dynamic structure mediates halophilic adaptation of a DNA polymerase from the deep-sea brines of the Red Sea

**DOI:** 10.1096/fj.201700862RR

**Published:** 2018-01-24

**Authors:** Masateru Takahashi, Etsuko Takahashi, Luay I. Joudeh, Monica Marini, Gobind Das, Mohamed M. Elshenawy, Anastassja Akal, Kosuke Sakashita, Intikhab Alam, Muhammad Tehseen, Mohamed A. Sobhy, Ulrich Stingl, Jasmeen S. Merzaban, Enzo Di Fabrizio, Samir M. Hamdan

**Affiliations:** * Biological and Environmental Science and Engineering Division, King Abdullah University of Science and Technology, Jeddah, Saudi Arabia;; † Physical Science and Engineering Division, King Abdullah University of Science and Technology, Jeddah, Saudi Arabia;; ‡ KAUST Catalysis Center, King Abdullah University of Science and Technology, Jeddah, Saudi Arabia;; § Computational Bioscience Research Center, King Abdullah University of Science and Technology, Jeddah, Saudi Arabia; and; ¶ Fort Lauderdale Research and Education Center, University of Florida, Davie, Florida, USA

**Keywords:** DNA polymerase engineering, halophilic enzymes, thermophilic enzymes, structure dynamism, structural adaptation

## Abstract

The deep-sea brines of the Red Sea are remote and unexplored environments characterized by high temperatures, anoxic water, and elevated concentrations of salt and heavy metals. This environment provides a rare system to study the interplay between halophilic and thermophilic adaptation in biologic macromolecules. The present article reports the first DNA polymerase with halophilic and thermophilic features. Biochemical and structural analysis by Raman and circular dichroism spectroscopy showed that the charge distribution on the protein’s surface mediates the structural balance between stability for thermal adaptation and flexibility for counteracting the salt-induced rigid and nonfunctional hydrophobic packing. Salt bridge interactions *via* increased negative and positive charges contribute to structural stability. Salt tolerance, conversely, is mediated by a dynamic structure that becomes more fixed and functional with increasing salt concentration. We propose that repulsive forces among excess negative charges, in addition to a high percentage of negatively charged random coils, mediate this structural dynamism. This knowledge enabled us to engineer a halophilic version of *Thermococcus kodakarensis* DNA polymerase.—Takahashi, M., Takahashi, E., Joudeh, L. I., Marini, M., Das, G., Elshenawy, M. M., Akal, A., Sakashita, K., Alam, I., Tehseen, M., Sobhy, M. A., Stingl, U., Merzaban, J. S., Di Fabrizio, E., Hamdan, S. M. Dynamic structure mediates halophilic adaptation of a DNA polymerase from the deep-sea brines of the Red Sea.

The deep-sea anoxic brines of the Red Sea are considered among the most remote, challenging, and extreme environments on earth. Approximately 25 such brine-filled pools have been discovered in the Red Sea (1–3), all of which are anoxic and highly saline deep-seawater pools with elevated temperatures and heavy metal ion concentrations. In contrast to numerous geological and geochemical studies of the brine pools, very few studies have focused on the microbiology of deep-sea brines (4). Novel culture-independent techniques, accompanied by traditional culture-based studies, have revealed an unexpected and enormous biodiversity in the local microbial communities of the brine pools and have identified several new groups of extremophilic microorganisms (5–7). The adaptive mechanisms of biologic macromolecules in these microorganisms remain uncharacterized. Given that DNA-binding proteins require positively charged residues to interact with the negative charges on DNA phosphate groups, we wondered how DNA-processing enzymes in these microorganisms interact with DNA at elevated salt concentrations.

Indeed, salt has significant effects on the solubility, stability, and conformation of proteins and is likely to influence their function (8, 9). Water hydrates surface-exposed charged residues to enhance protein solubility. In addition, water cages solvent-exposed hydrophobic patches, which play a critical role in folding the protein into its functional form (10). When present, salt strengthens hydrophobic packing and contributes to protein folding; however, elevated salt concentrations sequester water molecules from solvent-exposed hydrophobic patches, possibly promoting rigid and nonfunctional hydrophobic packing and protein aggregation (10, 11). Halophilic proteins may overcome such salt-induced hydrophobic packing by increasing the hydrophilicity and decreasing the hydrophobicity of their surfaces (12–15). Increasing the number of negatively charged residues (Asp and Glu) could improve the hydrophilicity of the surface. Asp and Glu have great capacity to bind water molecules and utilize salts by binding hydrated cations (12–18). Decreasing the number of hydrophobic residues with bulky side chains (Phe, Ile, and Leu) rather than those with small side chains (Gly, Ala, Ser, and Thr) might reduce surface hydrophobicity (14, 19). Salt bridges between surface-exposed, oppositely charged residues could also influence the stability and folding of halophilic proteins (20, 21). Collectively, these previous findings suggest that halophilic proteins may be overly flexible at low salt concentrations and consequently less active, which may explain their increased activity at higher salt concentrations.

The structural requirements of thermophilic proteins, conversely, are likely to be opposite those of halophilic proteins. Strong hydrophobic packing through hydrophobic residues with bulky side chains and salt bridge interactions between oppositely charged residues might be the key mechanisms that increase the stability of thermophilic proteins (22, 23). The simultaneous increase in both temperature and salt concentrations in the deep-sea brines in the Red Sea suggests that proteins in extremophilic microorganisms paradoxically adapt to 2 environments with opposite structural requirements.

Little is known about how halophilic DNA-binding proteins interact with DNA. Analysis of the surface charges combined with site-directed mutagenesis among halophilic and mesophilic transcription factors [TATA box–binding proteins (TBPs)] showed that halophilic TBP increases the number of negatively charged residues in the DNA-binding region. These charges may sequester positive ions into the DNA-binding region, allowing indirect interaction with the DNA *via* salt bridges. This indirect interaction could support DNA binding at elevated salt concentrations (24, 25). However, enzymes involved in genomic DNA replication and maintenance often experience conformational changes and induce changes in the DNA structure for catalysis. We therefore analyzed how indirect interactions with DNA support the functioning of halophilic DNA-binding enzymes, such as DNA polymerases, under hypersaline conditions.

Characterization of a polymerase, termed brine pool-3 polymerase (BR3 Pol), from archaea that thrive at temperatures approaching ∼55°C demonstrated unusual tolerance to high salt and metal ion concentrations and a unique ability to use Zn^2+^ ions effectively as cofactors. These observations suggested that structural adaptation is required in the proteins of brine pool microorganisms. The simultaneous adaptation to high temperature and high salt concentration is mediated by increasing salt bridge interactions that enhance structural stability, whereas dynamic fluctuation is maintained by a high percentage of negatively charged random coils. Functional and Raman analyses revealed that the structure becomes more fixed and functional with increasing salt concentration. We also found that the polymerase is likely to maintain the conserved direct interaction mode with DNA. Our results suggest that catalysis in halophilic DNA polymerases relies on direct DNA interactions, whereas the structural flexibility that counteracts salt-induced, rigid, and hydrophobic packing allows active conformation in a wide range of salt concentrations.

## MATERIALS AND METHODS

### Enzymes

We deposited the cDNA fragment corresponding to BR3 Pol in GenBank under accession number KXB03331. To amplify the open reading frame (ORF) region by PCR [*Thermococcus kodakarensis* (KOD) polymerase], single amplified genomic DNA samples were used as templates with primers (5′-CACCATGGCAAATCAGACAACAAATGG-3′ and 5′-TTATTTGAATTTTCCGAGTTTTACTTGTCG-3′) (26). The thermal cycler program used for PCR reactions consisted of a predenaturing step (94°C for 2 min), a denaturing step (94°C for 15 s), an annealing step (56°C for 30 s), and an extension step (68°C for 2 min). The denaturing to extension steps were repeated 29 times. The resulting clone was sequenced and showed 9-aa insertion at N terminus compared with the published sequence in the database. We added this insertion to the ORF and cloned it into the pENTR/D-TOPO vector (Thermo Fisher Scientific, Waltham, MA, USA). The ORF of BR3 Pol identified from the single amplified genome was transferred to the pDEST17 vector (Thermo Fisher Scientific) by using the LR Clonase II enzyme mix (Thermo Fisher Scientific). BR3 Pol was overexpressed in *Escherichia coli* Rosetta 2(DE3) (Novagen, Stamford, CT, USA) and transformed with the pDEST17/BR3 plasmid. Overexpression was induced by the addition of isopropyl β-D-1-thiogalactopyranoside (final concentration, 1 mM), and cells were harvested after 3 h of incubation.

The collected cells were resuspended in lysis buffer (10 mM Tris-HCl pH 8.0, 80 mM KCl, 5 mM 2-ME, 1 mM EDTA) and incubated on ice with lysozyme (final concentration, 1 mg/ml) for 60 min and then disrupted by sonication. The crude extract was centrifuged to remove cell debris, and the collected supernatant was subjected to ammonium sulfate precipitation at 70% saturation. The pellet obtained from ammonium sulfate precipitation was dissolved in Buffer A (50 mM Tris-HCl pH 8.0, 500 mM NaCl) and loaded onto a Sephacryl Sepharose column (GE Healthcare, Waukesha, WI, USA). The flow-through fractions containing BR3 Pol were collected and loaded into a HisTrap HP 5 ml column (GE Healthcare) in which a gradient against Buffer B (10 mM Tris-HCl pH 8.0, 50 mM KCl, 500 mM imidazole) eluted the bound proteins. The BR3 Pol–containing fractions were collected and passed through a HiTrap Heparin 1 ml column (GE Healthcare) in which a gradient against Buffer C (10 mM Tris-HCl pH 8.0, 1 M KCl) eluted the bound proteins. The BR3 Pol–containing fractions were dialyzed against Buffer D (50 mM Tris-HCl pH 7.5, 50 mM KCl, 1 mM DTT, 0.1% Tween 20, 50% glycerol). The protein concentration was determined by absorbance at 280 nm. ProtParam software (Swiss Institute of Bioinformatics, Lausanne, Switzerland; *https://web.expasy.org/protparam/* was used to calculate the extinction coefficient of BR3 Pol based on the amino acid sequence composition (27).

pCold/KODWT plasmid to express N-terminal his-tagged KOD wild type (WT) was kindly given by Dr. Takahiro Kusakabe (Kyushu University, Fukuoka, Japan). KOD_BR3exo_, KOD_BR3fingers_, and KOD_BR3thumb_ were constructed by using Gibson Assembly (New England Biolabs, Ipswich, MA, USA). The primers for KOD and the exonuclease domain of BR3 assembly to make KOD_BR3exo_ were BR3exo1: 5′-CAAAGGTCTGGTTCCGATGGAAGCAGACGAAGAACTCAATATCC-3′; BR3exo2: 5′-AGCTGAGCTTCCATCGGCAGTATTTCAGTGCCTAGCTCTAGAGTTG-3′; KOD1: 5′-TTCCATCGGAACCAGACCTTTG-3′; and KOD2: 5′-CTGCCGATGGAAGCTCAGCTGTC-3′. The primers for KOD and the fingers domain of BR3 assembly to make KOD_BR3fingers_ were BR3fingers1: 5′-GTTTCTGCAAAGACTTCCCGGGTTTCATTCCGGAGACATTGAAAG-3′; BR3fingers2: 5′-GCAGTACCAACGAGCACGAGCGTATCCAAGCATCCCATAGAATGAGTTC-3′; KOD3: 5′-CGGGAAGTCTTTGCAGAAACG-3′; and KOD4: 5′-GCTCGTGCTCGTTGGTACTG-3′. The primers for KOD and the thumb domain of BR3 assembly to make KOD_BR3thumb_ were BR3thumb1: 5′-GAAGGTTTCTACAAGCGTGGCGTATTCGTCACCAAAAAAAGATACGCAATG-3′; BR3thumb2: 5′-TGGGTCGGCGCGCCCACCCTTTTATTTGAATTTTCCGAGTTTTACTTGTCGC-3′; KOD5: 5′-GCCACGCTTGTAGAAACCTTC-3′; and KOD6: 5′-AAGGGTGGGCGCGCCG-3′.

The KOD chimera proteins were overexpressed in *E coli* BL21(DE3) pLysS (Novagen). The transformed cells were incubated at 37°C to grow, and after cooling down the temperature to 16°C, isopropyl β-D-1-thiogalactopyranoside (final concentration, 1 mM) was added to induce the overexpression. The cells were incubated at 16°C for 6 h and harvested. The collected cells were dissolved in lysis buffer (10 mM Tris-HCl pH 8.0, 80 mM KCl, 5 mM 2-ME, 1 mM EDTA) and incubated on ice with lysozyme (final concentration, 1 mg/ml) for 60 min and then disrupted by sonication. The crude extract was centrifuged to remove cell debris, and the collected supernatant was subjected to ammonium sulfate precipitation at 70% saturation. The pellet obtained from ammonium sulfate precipitation was dissolved in Buffer A (50 mM Tris-HCl pH 8.0, 500 mM NaCl) and loaded into a HisTrap FF 5-ml (GE Healthcare) in which a gradient against Buffer B (10 mM Tris-HCl pH 8.0, 50 mM KCl, 500 mM imidazole) eluted the bound proteins. The KOD chimera protein-containing fractions were collected and passed through a HisTrap Heparin 1-ml column (GE Healthcare) in which a gradient against Buffer F (20 mM Tris-HCl pH 8.0, 10 mM 2-ME, 1 M NaCl) eluted the bound proteins. The KOD chimera protein-containing fractions were dialyzed against Buffer G (50 mM Tris-HCl pH 8.0, 0.1 mM EDTA, 0.1 mM DTT, 0.1% NP-40, 0.1% Tween 20, 50% glycerol). The protein concentration was determined by absorbance at 280 nm using the extinction coefficient and MW calculated based on the amino acid sequence composition of KOD chimera proteins by using ProtParam software (27).

### Predictions of disordered regions in proteins

The probability of disorder was calculated by using Regional Order Neural Network (RONN) software (*https://www.strubio.ox.ac.uk/RONN*) (28–30).

### Raman spectroscopy

Five-microliter solutions of each sample were drop-casted on CaF_2_ substrates (Crystran, Poole, United Kingdom) and investigated with a WITec confocal Raman system (WITec Instruments, Knoxville, TN, USA), equipped with a 532-nm laser and a ×100 objective, working at a laser power of 1.32 mW with a 600 line/mm grating. The measurements were conducted in the back-scattering configuration and performed in the liquid state. Data analysis was performed by using ∼10 spectra obtained on different points of the droplet. Measurements were performed at room temperature (21°C) and 53% relative humidity. Data analysis was performed by using WITec Project 4 software (WITec Instruments.).

### Circular dichroism spectroscopy analysis

Circular dichroism (CD) measurements were performed at wavelengths from 200 to 250 nm at 20°C using 1 nm intervals with 10 accumulations per read. The proteins were diluted to a final concentration of 3 µM (BR3 Pol) and 1.3 µM (KOD Pol) in PBS buffer containing the corresponding salt concentrations and incubated at room temperature for 5 min before the measurements. A sample volume of 300 µl was used in a 0.1-cm quartz cuvette (Hellma, Huntington, NY, USA), and the measurement was performed on a Jasco J-815 Spectrometer (Jasco, Easton, MD, USA). The molar ellipticity was calculated by Spectra Manager II (Jasco).

### Primer extension assays

The polymerase activity was measured by using primer extension assays, which were conducted as previously described (31) with the following modifications. A 35-mer template was annealed to a 15-mer Cy3-labeled primer. The primer extension assays were performed in a reaction buffer containing 20 mM Tris-HCl pH 8.8, 10 mM (NH_4_)_2_SO_4_, 0.1% Triton X-100, 200 µM deoxynucleotide triphosphates (dNTPs), 50 mM KCl, and 2 mM MgSO_4_ at 45 C° for 4 min and stopped with EDTA (final concentration, 100 mM pH 8.0). The same reaction buffer without salt and/or metal ions was used for the assays with various types and concentrations of salts and metal ions as specified in the figure legends. The reactions were terminated by EDTA (final concentration, 100 mM pH 8.0). The synthesized products were loaded on 20% polyacrylamide/7.5 M urea/1× TBE denaturing gel, and the gel was visualized by using Typhoon Trio (GE Healthcare). Each experiment was performed at least 3 times to confirm reproducibility.

### Single-molecule primer extension assays

The primed single-stranded DNA (ssDNA) was generated by annealing M13mp18 ssDNA (New England Biolabs, Ipswich, MA, USA) to 100-fold excess of the 5′-Dig-CTAGAGGATCCCCGGGTACCGAGCTCGAATTCGTAATCA-Bio-TGGTCATAGCTGTTTCCTGTGTG-3′ primer (Integrated DNA Technologies, Coralville, IA, USA). Annealed DNA was linearized with *Eco*RI (New England Biolabs), and the reaction was stopped by EDTA (final concentration, 10 mM pH 8.0). *Eco*RI was inactivated by heating the solution at 65°C for 20 min. Excess unannealed primer and heat-inactivated *Eco*RI were removed by using a 100 kD Amicon centrifugal filter device (EMD Millipore, Billerica, MA, USA). The final concentration of the DNA was quantified by using UV-visible absorption spectroscopy at 260 nm with an extinction coefficient of 91,801.1 mM/cm.

Single-molecule experiments were performed at 37°C in a custom-built microfluidic flow cell as described previously (32, 33). Briefly, *Pyrococcus furiosus* (Pfu) Pol and BR3 Pol were introduced into the flow cell at 20 nM concentration in a reaction buffer containing 20 mM Tris-HCl pH 7.5, 5 mM MgCl_2_, 5 mM DTT, and 760 µM dNTPs, with 250 mM KCl for BR3 Pol and 0 mM KCl for Pfu Pol. The replication reaction was performed under continuous presence of proteins in solution. Data acquisition and processing methods were essentially the same as described previously (34–36). Briefly, the centroid position of the beads during each acquisition time point (500-ms sampling rate) was determined by fitting a 2-dimensional gaussian distribution of the bead intensities by using the DiaTrack particle-tracking software (SemaSopht, North Epping, NSW, Australia). Residual instabilities in the flow were corrected by subtracting traces corresponding to tethered DNA molecules that were not enzymatically altered from all traces. Displacement of the beads due to the conversion of the template strand from ssDNA to double-stranded DNA (dsDNA) during primer extension was converted into numbers of synthesized nucleotides by using a calibration factor (3.76 b/nm) that was derived from the difference in the length between ssDNA and dsDNA at the applied stretching force (∼3 pN) (35–37). Pauses in DNA synthesis were defined as a minimum of 6 data points with no change in the DNA length with amplitude fluctuations <3 times the sd of the noise.

### Proofreading activity assays

The proofreading activity assays were conducted as previously described (31) with the following modifications. A 35-mer template containing an internal *Eco*RI site was annealed to a 15-mer Cy3-labeled primer with 0, 1, 2, or 3 mismatched nucleotides at the 3′ end for the proofreading assays (Supplemental Fig. 8*A*). The proofreading assays were performed in a reaction buffer containing 20 mM Tris-HCl pH 8.8, 10 mM (NH_4_)_2_SO_4_, 0.1% Triton X-100, 200 µM dNTPs, and 2 mM MgSO_4_ at 45 C° for 4 min and stopped with EDTA (final concentration, 100 mM pH 8.0). The reaction products were denatured, and EDTA was removed; 5 U of *Eco*RI was then added, followed by additional incubation at 37°C for 30 min. The reactions were terminated by EDTA (final concentration, 100 mM pH 8.0). The synthesized products in the proofreading assays were loaded on 20% polyacrylamide/7.5 M urea/1× TBE denaturing gel, and the gel was visualized by using Typhoon Trio (GE Healthcare).

### PCR reactions

The PCR reactions were performed in a reaction buffer containing 20 mM Tris-HCl pH 7.5, 500 µM dNTPs, 2 mM MgCl_2_ with various concentrations of NaCl and using 5 nM protein concentration. pUC19 vector (New England Biolabs) was used as a template to amplify the ampicillin resistance gene with the following primers: 5′-TTACCAATGCTTAATCAGTGAGGCACC-3′ and 5′-ATGAGTATTCAACATTTCCGTGTCGCCC-3′. The thermal cycler program used for PCR reactions consisted of a predenaturing step (94°C for 2 min), a denaturing step (94°C for 15 s), an annealing step (56°C for 30 s), and an extension step (68°C for 1 min). These steps were repeated 29 times.

### Surface plasmon resonance binding

Surface plasmon resonance (SPR) binding was performed by using a Biacore T100 label-free system (GE Healthcare). The system was washed twice with 1× HBS-EP buffer before DNA immobilization. The DNA constructs used in the analysis were ssDNA (5′-/Biotin/TTTTTTTTTTTTTTTTTTTTTTTTTTTTTTTTTTTTTTTT-3′), primer/template strands (a template 5′-GAGGTGGCGTCGGGTGGACGGGTGGATTGAAATTTAGGCTGGCAC/Biotin/-3′ annealed to 5-fold excess of non-biotinylated primer 5′-GTGCCAGCCTAAATTTCAATCC-3′), and dsDNA (the same template as in the primer/template strands DNA construct but annealed to 5-fold excess of non-biotinylated oligo 5′-GTGCCAGCCTAAATTTCAATCCACCCGTCCACCCGACGCCACCTC-3′). Annealing was performed in a buffer containing 20 mM Tris-HCl pH 7.0, 50 mM NaCl by heating at 90°C for 3 min and slowly cooling down to room temperature. A streptavidin sensor chip was activated by 3 injections of 50 mM NaOH in 1 M NaCl followed by immobilizing the biotinylated DNA constructs at a flow rate of 10 µl/min. The number of response units (RUs) for the immobilized DNA constructs was maintained between 75 and 150 depending on the MW of each substrate to ensure that the maximum RU from protein binding was <500. A control flow cell that was activated and blocked in the absence of protein or DNA was used to subtract the RU resulting from the nonspecific interactions with the sensor chip and the buffer’s bulk refractive index. Before conducting the binding study, the system was primed twice with the corresponding binding buffer (20 mM Tris-HCl pH 8.8, 10 mM [NH_4_]_2_SO_4_, 0.1% Triton X-100, and the desired NaCl concentrations). In the binding study, serial dilutions of BR3 Pol, KOD Pol WT, KOD_BR3exo_, and KOD_BR3thumb_ were made using the corresponding binding buffer. At each concentration, the run started with a surface-regeneration injection of the binding buffer in the presence of 1 M NaCl at a flow rate of 100 µl/min for 120 s, followed by protein sample injection at a flow rate of 20 µl/s for 70 s. The corrected sensorgrams were processed by using Biacore T100 Evaluation Software (GE Healthcare). The maximum RU reached at each protein concentration was fitted by using the steady-state affinity mode to obtain the equilibrium dissociation constant (*K_d_*) for each DNA substrate.

## RESULTS

### Primary sequence characterization of BR3 Pol

**Figure 1.**
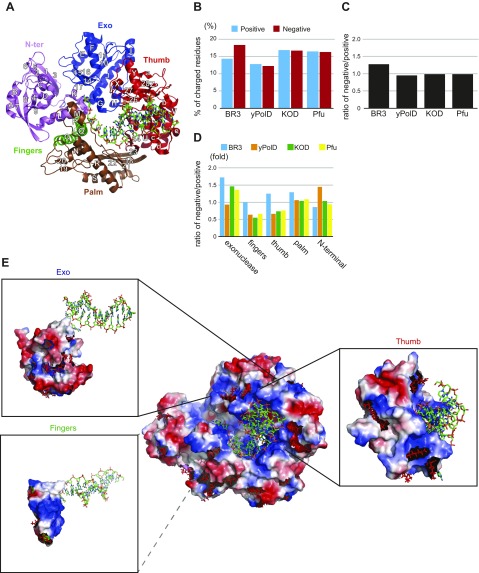
Primary sequence analysis of BR3 Pol. *A*) Structure of KOD Pol bound to DNA in the polymerization mode. The protein is depicted as a cartoon and DNA as sticks (PDB ID: 4K8Z) (46). The 5 domains in the polymerase and their secondary structure elements are colored as indicated. *B*) Traits of positively and negatively charged residues in BR3 Pol compared with the catalytic subunit of yPolD and to the archaeal polymerases KOD Pol and Pfu Pol. Percentages of positively charged residues (basic residues: Arg and Lys) and negatively charged residues (acidic residues: Glu and Asp) in BR3 Pol, yPolD, KOD Pol, and Pfu Pol are shown. *C*) Ratio of negatively charged to positively charged residues in BR3 Pol, yPolD, KOD Pol, and Pfu Pol. *D*) Ratio of negatively charged to positively charged residues for the 5 domains in the polymerase in BR3 Pol, yPolD, KOD Pol, and Pfu Pol. *E*) Molecular surface with electrostatic potential map in KOD Pol. The red and blue surfaces are acidic (negatively charged) and basic (positively charged) residues, respectively. The residues on the surface of KOD Pol that were replaced with unique acidic residues (Glu or Asp) in BR3 Pol on the surface are shown as red sticks. The electrostatic potential maps of the exonuclease, fingers, and thumb domains with DNA and the locations of unique acidic residues in BR3 Pol in each domain are highlighted.

The archaeal species harboring BR3 Pol was identified from a water layer in the deep-sea brines with a temperature of ∼55°C and a salt concentration of ∼2.6 M (unpublished results). We thus expected BR3 Pol to be both thermophilic and extremely halophilic. Sequence alignment of the dedicated 818 aa of BR3 Pol identified in INDIGO (6) with 3 extremely thermophilic archaeal DNA polymerases, KOD Pol, Pfu Pol, and *Thermococcus litoralis* (also known as Vent Pol), showed a high similarity of ∼45% (Supplemental Fig. 1). We therefore concluded that BR3 Pol is also a member of the family B DNA polymerases. Guided by the crystal structures of KOD Pol and Pfu Pol (38, 39), we defined 5 domains in BR3 Pol, including the N-terminal, exonuclease, palm, fingers, and thumb domains (**Fig. 1*A***), which constitute the conserved and partially closed right-hand feature that binds the primer/template strands.

The domains in BR3 Pol are largely conserved but display variation in the percentage of charged residues. Increasing the percentage of both negatively (Asp and Glu) and positively (Arg and Lys) charged residues to promote salt bridge interactions is a common adaptive mechanism in proteins from extreme thermophiles as well as in those from halophiles (10, 15, 20). We found this scenario to be the case in BR3 Pol as well as in KOD Pol and Pfu Pol compared with a family B mesophilic polymerase represented here by the catalytic subunit of yeast DNA polymerase δ (yPolD) (Fig. 1*B*). However, unlike KOD Pol and Pfu Pol, which maintain a similar ratio of negatively-to-positively charged residues as that in yPolD, BR3 Pol has more negatively charged residues (Fig. 1*B, C*). This outcome is consistent with the observation that halophilic proteins tend to have more negatively charged residues than mesophilic proteins (13, 15, 18, 19, 40, 41). Inserts with high Asp and/or Glu contents are a common feature in halophilic proteins (15, 42–44). In BR3 Pol, most acidic residues are distributed across the protein (Fig. 1*D* and Supplemental Fig. 2) despite one of its two unique inserts being highly acidic (Supplemental Fig. 1).

Because there is no clear increase in the number of hydrophobic amino acids with large or small side chains over the number of such amino acids in mesophilic yPolD (Supplemental Fig. 2*A*), the contribution of hydrophobic packing to thermal stability in KOD Pol, Pfu Pol, and BR3 Pol is unclear. Disulfide bonds could be a key adaptive feature in extremely thermostable polymerases (15, 45). BR3 Pol retained its activity in temperatures up to ∼55°C (Supplemental Fig. 3*A*), and it is therefore classified as a thermophilic and not as an extremely thermophilic protein. BR3 Pol contains highly conserved Cys residues in the palm domain that have been proposed to create a disulfide bond in extremely thermophilic DNA polymerases (Supplemental Fig. 1) (38). KOD Pol and Pfu Pol, but not the extremely thermophilic polymerase *T*. *litoralis* Pol, have an extra disulfide bond in their palm domain. Introducing an equivalent disulfide bond in BR3 Pol, however, did not improve the protein activity at higher temperatures. Collectively, these results suggest that salt bridge interactions could be the major contributor to thermal stability in BR3 Pol. Salt tolerance, conversely, is likely mediated by excess negatively charged residues and structural stabilization *via* salt bridge interactions.

### Distribution of charged residues on the surface of BR3 Pol

DNA polymerases from the B family, such as KOD Pol and Pfu Pol, have significant structural similarities (38, 39, 46). We found high sequence homology between BR3 Pol and KOD Pol (∼45%). We also found that chimera proteins of KOD Pol containing exonuclease, fingers, or thumb domains of BR3 Pol displayed normal polymerization activity (as discussed later), suggesting that BR3 Pol is likely to have a high structural similarity to KOD Pol. We thus performed sequence alignment between BR3 Pol and KOD Pol and used the structure of KOD Pol bound to the DNA in the polymerization mode to provide reliable predictions on the location of the negatively and positively charged residues in BR3 Pol. We noticed that unique negatively charged residues in the fingers, exonuclease, and thumb domains of BR3 Pol would be positioned away from the DNA-binding crevice and toward its outer surface (Fig. 1*E* and Supplemental Fig. 4). The thumb domain of KOD Pol contains 20 residues that are within 3.6 Å of the DNA. Almost all of these residues are conserved in BR3 Pol (Supplemental Fig. 1). These observations suggest that the DNA-binding crevice is well conserved in BR3 Pol and that the increased negatively charged residues are primarily distributed across its surface.

### BR3 Pol has increased flexible regions

**Figure 2.**
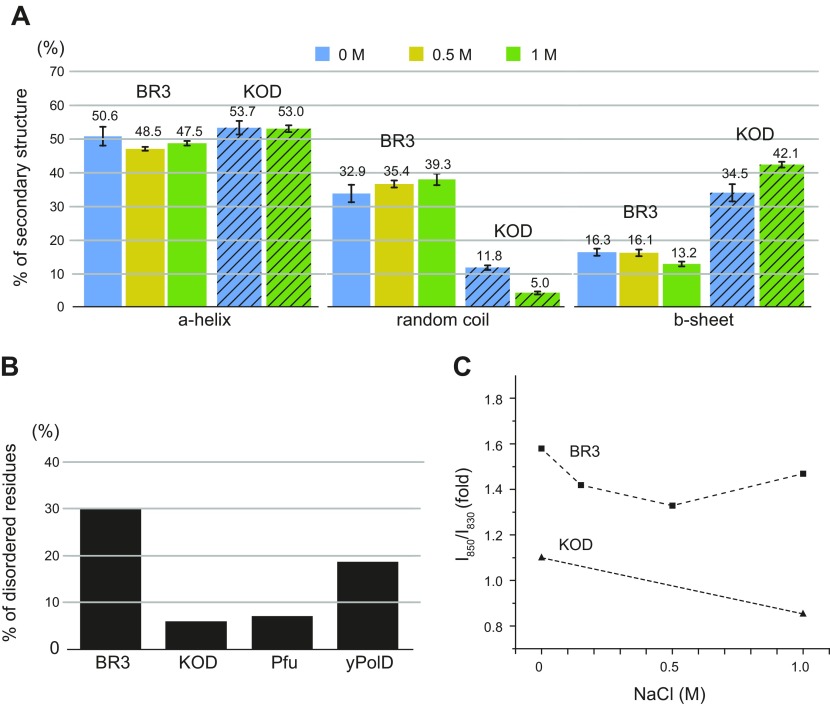
Protein structure analysis of BR3 Pol and KOD Pol. *A*) Percentages of α-helices, random coils, and β-sheets in BR3 Pol (solid) and in KOD Pol (hatched) analyzed by using Raman spectroscopy. Supplemental Fig. 5*A* illustrates fitting of the secondary structure region. The numbers above each bar indicate the value in a percentage, and sd is shown. *B*) Percentage of amino acids identified as a disordered region in BR3 Pol, KOD Pol, Pfu Pol, and yPolD. Disorder probability was calculated by RONN (28), and plots of disorder probability are presented in Supplemental Fig. 6*A*. *C*) Hydroxyl group interaction with hydrogen bonds of BR3 Pol and KOD Pol as a function of NaCl concentration. Supplemental Fig. 5*B* illustrates curve fitting of the doublet of the Tyr band.

We next obtained secondary structure information on purified recombinant BR3 Pol by using Raman spectroscopy. These measurements were performed with the protein in solution to maintain its native state. The secondary structure was estimated by fitting the spectral region of 1500–1800 cm^−1^ with 6 components of gaussian-lorentzian mixed functions. The bands centered at ∼1550, 1580, 1610, 1650, 1670, and 1680 cm^−1^ are associated with the indole ring of Trp, the combination of alanine and glycine, the combination of Tyr, Trp, and Phe, the α-helix, the random coil and the β-sheet, respectively (47–49). Fitting of the secondary structure region of BR3 Pol with varying NaCl concentrations is shown in Supplemental Fig. 5*A*. With no salt, BR3 Pol contains a high percentage of random coils (∼30%) (**Fig. 2*A***). In a side-by-side comparison, KOD Pol had 3-fold fewer random coils than BR3 Pol. These results are consistent with the prediction of intrinsic disorder by the RONN software (28–30), which showed an ∼30% disordered region in BR3 Pol (Fig. 2*B* and Supplemental Fig. 6*A*). CD analysis also indicate that BR3 Pol has more random coil and β-sheet than KOD Pol because it has the less pronounced local minimum at 222 nm, and its signal is upshifted between 208 and 222 nm compared with KOD Pol (Supplemental Fig. 6*B*) (50). RONN also predicted that BR3 Pol has the most disordered regions, followed by the mesophilic protein yPolD and then the extremely thermophilic proteins Pfu Pol and KOD Pol (Fig. 2B). We propose, therefore, that adaptation in halophilic proteins requires a flexible structure as opposed to the more rigid structure needed by extremely thermophilic proteins.

RONN analysis showed that the disordered regions in BR3 Pol were distributed across the entire protein (Supplemental Figs. 1 and 6*A*). Prediction of their locations based on the KOD Pol structure suggests that they should primarily be positioned on the protein’s surface (Supplemental Figs. 1 and 4). Interestingly, RONN analysis suggested that 63 and 36% of the unique Asp and Glu residues, respectively, in BR3 Pol should be in these disordered regions (Supplemental Fig. 6*C*). It is unclear how Asp and Glu residues influence protein structure, but it has been suggested that Asp tends to act as an α-helix breaker (51, 52) and exists more than Glu in the coiled regions of proteins (53). Halophilic proteins tend to have more Asp than Glu residues (54). In support of this suggestion, we found a strong correlation between the number of unique Asp residues and their presence in the disordered regions of BR3 Pol. We also suggest that the increased negative charges by both Asp and Glu residues may enhance the overall structural flexibility through repulsive forces.

### Salt induces transitioning in BR3 Pol from a dynamic to a more defined structure

Raman spectroscopy was next used to investigate the effect of salt on the structure of BR3 Pol compared with KOD Pol. With increased NaCl concentration, we found that BR3 Pol and KOD Pol maintained their overall secondary structure composition (Fig. 2*A*). The CD spectrum of BR3 Pol and KOD Pol at different salt concentrations confirmed that minor changes occur in their secondary structures with elevated NaCl concentrations (Supplemental Fig. 6*B*). These results show that BR3 Pol is structurally adapted to resist hydrophobic packing by remaining flexible in a wide range of salt concentrations.

We next obtained structural information on hydroxyl group interactions by monitoring the hydrogen-bonding state of the phenolic OH group in BR3 Pol and KOD Pol at different NaCl concentrations by following the ratio of intensities of the Tyr doublet (*I*_850_/*I*_830_). The ratio of intensities of the exposed Tyr doublet is a unambiguous marker of the hydrogen-bonding state of the phenolic OH group (55). *I*_850_/*I*_830_ for a solvent-exposed Tyr that forms hydrogen bonds with water is usually between 0.9 and 1.5, whereas *I*_850_/*I*_830_ for a buried Tyr within the protein network that forms hydrogen bonds with Asp and Glu residues is 0.7–1.0 (56). The ratio was calculated by integrating 2 peaks centered at 830 and 850 cm^−1^ in the spectral region of 800–880 cm^−1^ (Supplemental Fig. 5*B*); the same fitting criteria were used for KOD Pol. Overall, the phenolic hydroxyl groups of Tyr residues in BR3 Pol were stronger hydrogen bond acceptors than those in KOD Pol at all tested NaCl concentrations (Fig. 2*C*). This finding indicates that BR3 Pol can retain more water molecules than KOD Pol, presumably through its excess negatively charged residues that could interact directly with water and/or hydrated Na^+^ ions. The hydroxyl group interactions in BR3 Pol, however, decreased when the NaCl concentration was increased to 0.5 M before they started to increase again at an NaCl concentration of 1 M. Because the secondary structure was minimally influenced by salt, we interpreted the decreasing phase between 0 and 0.5 M NaCl concentrations as reflecting a transition from a highly flexible and dynamic structure that rapidly exchanges water to a more defined structure that binds water and/or hydrated Na^+^ ions in fixed positions. The increasing phase in the hydroxyl group interaction between 0.5 and 1 M NaCl concentration is more difficult to explain. We suspect that it might reflect the exchange of saturated protein-bound hydrated Na^+^ ions with ions from the solution and/or from an increase in the dielectric constant of the excess negatively charged residues. The hydroxyl group interaction in KOD Pol, conversely, was weaker at 1 M than at the no salt condition. Together, these results suggest that salt may induce rigid structural transitioning in KOD Pol as opposed to its dynamic structural transitioning in BR3 Pol.

### BR3 Pol tolerates high salt concentrations

**Figure 3.**
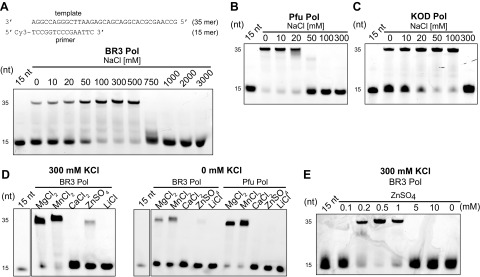
Dependence of the polymerase activity on salt and metal ions. *A*) Salt-dependent polymerization activity of BR3 Pol. Polymerase activity was measured on a short primer/template substrate consisting of a 15-mer primer labeled at its 5′ end with Cy3 and a 35-mer template strand (insert schematic). Reactions were performed in a buffer containing the indicated concentration of NaCl for 4 min at 45°C and stopped by EDTA, as described in the Materials and Methods section. Products were analyzed on a denaturing 20% polyacrylamide urea gel. *B*, *C*) Salt-dependent polymerization activity of Pfu Pol and KOD Pol, respectively. *D*) Effect of different metal ions on the polymerization activity of BR3 Pol compared with Pfu Pol. Different metal ions were added at a constant concentration of 2 mM with the reaction performed in a buffer containing either 300 mM KCl (left panel) or 0 mM KCl (right panel). *E*) Effect of increasing ZnSO_4_ concentration on the polymerization activities of BR3 Pol in a reaction buffer containing 300 mM KCl. Reactions in *B*–*E* were performed and analyzed as in *A*.

We next characterized the effect of salt on the polymerization activity of BR3 Pol and directly compared it with purified KOD Pol and Pfu Pol. The activity of BR3 Pol gradually increased with increasing salt concentration and approached its maximum at 0.5 M NaCl before it quickly declined at 0.75 M (**Fig. 3*A***). This greater activity upon increased salt concentration is consistent with the activity observed in halophilic proteins (18, 57). The optimal activity at 0.5 M NaCl also supports our proposition that the lowest hydroxyl group interaction at 0.5 M NaCl might reflect a more defined and functional structure. Nonetheless, this structure must still have dynamic fluctuations at 0.5 M to meet the required conformational changes during catalysis. In fact, the hydroxyl group interaction of BR3 Pol at 0.5 M was even higher than that of KOD Pol (Fig. 2*C*).

Pfu Pol and KOD Pol were more active than BR3 Pol at lower salt concentrations, but their activity declined dramatically beyond 20 and 100 mM NaCl, respectively (Fig. 3*B*, *C*). BR3 Pol also displayed remarkable resistance to increasing concentrations of a variety of salts (Supplemental Fig. 3*B*). The trend in polymerase activity in different salt concentrations among these polymerases reflects the trend in the external salt concentrations of their harboring archaea species (58, 59), suggesting that the macromolecules of archaea from the deep-sea brines in the Red Sea, as well as their membrane transport mechanisms, adapt to the intracellular salt concentration (18, 60).

**Figure 4.**
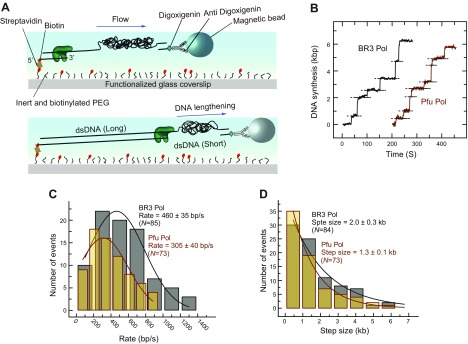
Single-molecule measurements of rate and processivity of BR3 Pol and Pfu Pol. *A*) Schematic depiction of the single-molecule assay for observing the primer extension reaction. *B*) Representative primer extension trajectories by BR3 Pol and Pfu Pol. Dotted lines indicate pausing of the primer extension, which represents points where a minimum of 3 s of no change in DNA length was detected. *C*) Distribution of rate of DNA synthesis by BR3 Pol and Pfu Pol extracted from the slope of the DNA lengthening phase in *B* fitted with a gaussian distribution. *D*) Distribution of processivity of DNA synthesis by BR3 Pol and Pfu Pol, extracted from the magnitude of the DNA lengthening phase in *B* fitted with a single exponential decay. Reactions were performed in a buffer containing 250 mM KCl for BR3 Pol and 0 mM KCl for Pfu Pol. The uncertainty corresponds to the sd of the fit.

We also measured the rate and processivity of BR3 Pol by a well-established single molecule replication assay that monitors DNA synthesis by measuring the change in the length of flow-stretched individual DNA molecules (33). Briefly, a ∼7 kbp ssDNA template was attached from 1 end to the surface of a glass coverslip of a microfluidic flow cell and from the other end to a magnetic bead (**Fig. 4*A***) and extended by a laminar flow exerting a ∼3 pN drag force on the beads. Primer extension converts the template strand from ssDNA (short) to dsDNA (long), causing an overall lengthening of the surface-tethered DNA molecules. The single molecule replication trajectories, at optimal 250 mM KCl, showed that the DNA synthesis segment was interrupted by pauses with no DNA synthesis (Fig. 4*B*). These pauses likely represent dissociation and reassociation events of the polymerases from solution because the polymerases are continuously present in the solution. The DNA synthesis phase would therefore represent the intrinsic rate and processivity of the DNA polymerase. By averaging over multiple segments (*n*) of the DNA synthesis phase (*n* = 83), the rate and processivity of BR3 Pol were found to be 460 ± 35 base/s and 2.00 ± 0.03 kb, respectively. In comparison, the rate and processivity of Pfu Pol, at optimal 0 mM KCl, were 305 ± 40 base/s (*n* = 73) and 1.30 ± 0.10 kb (Fig. 4*C, D*). These data show that BR3 Pol is ∼1.5-fold more active than Pfu Pol. The rate of Pfu Pol is significantly higher than previously reported in the literature using bulk replication assays (61). This outcome is likely due to including the pause duration in bulk replication assays, which would result in reporting slower apparent rates (Supplemental Fig. 7*A*, *B*). Bulk exonuclease proofreading assays, as described in the Materials and Methods section, showed that BR3 Pol had ∼2-fold higher proofreading activities than Pfu Pol did at their corresponding optimal salt concentrations (Supplemental Fig. 8*A–D*).

### BR3 Pol can effectively use Zn^2+^ ions as cofactors

The brine pools contain high concentrations of a variety of metal ions, including heavy metals (1). At an optimal high salt concentration (300 mM KCl) and a fixed concentration of metal ions (2 mM), BR3 Pol mediated extensive DNA synthesis using Mg^2+^, the most desired metal ion by DNA polymerases, as well as Mn^2+^, a popular alternative metal ion (62) (Fig. 3*D*). Surprisingly, we also detected weaker activity of BR3 Pol in the presence of Zn^2+^ ions but not in the presence of Li^+^ ions. Similar results were observed at the suboptimal salt concentration (0 mM KCl). In a control experiment, we showed that Pfu Pol was also most active in the presence of Mg^2+^ and Mn^2+^, although, in contrast to BR3 Pol, it was completely inactive in the presence of Zn^2+^ ions. Varying the concentration of Zn^2+^ ions showed that BR3 Pol activity could be dramatically increased at concentrations below 1 mM (Fig. 3*E*), approaching the activity measured in the presence of Mg^2+^ and Mn^2+^. These results suggest that BR3 Pol can effectively align the primer/template strands and the incoming dNTP *via* Zn^2+^ ions in its active site in contrast to other DNA polymerases. In the absence of salt, BR3 pol could use Mg^2+^ or Mn^2+^ as alternative sources of salt and could tolerate these metal ions at higher concentrations than could KOD Pol and Pfu Pol (Supplemental Fig. 3*C*, *D*). Collectively, these results show that BR3 Pol exhibits an outstanding tolerance to elevated MgCl_2_ and MnCl_2_ and an unusual ability to effectively utilize Zn^2+^ ions.

### Engineering a halophilic version of KOD Pol

**Figure 5.**
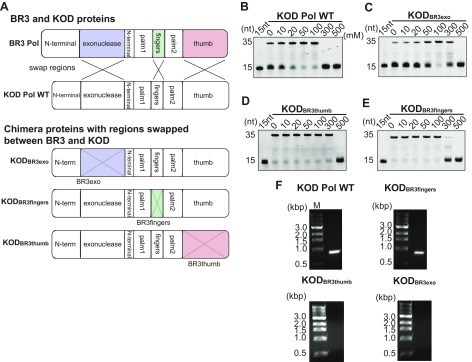
Salt-dependent polymerization activity of KOD Pol and its chimera proteins. *A*) Schematic of KOD Pol WT and chimeric proteins constructed between KOD Pol and BR3 Pol. The exonuclease, fingers, and thumb domains of KOD Pol swapped with those from BR3 Pol are shown in purple, green, and red, respectively. *B*–*E*) are the polymerization activities at increasing NaCl concentrations for KOD Pol WT, KOD_BR3exo_, KOD_BR3thumb,_ and KOD_BR3fingers_. The reaction was conducted and the product was analyzed as described in Fig. 3*A*. *F*) PCR test of KOD chimera proteins. PCR reactions were performed as described in the Materials and Methods section by KOD Pol WT, KOD_BR3fingers_, KOD_BR3thumb_, and KOD_BR3exo_.

We next investigated which regions in BR3 Pol are responsible for its salt tolerance. Due to the absence of a BR3 Pol structure, we relied on the strategy of replacing regions in KOD Pol with their corresponding homologous regions in BR3 Pol. Chimera proteins were constructed by swapping the exonuclease, fingers, and thumb domains of BR3 Pol with those in KOD Pol as illustrated in **Fig. 5*A***. We found that KOD_BR3exo_ and KOD_BR3thumb_ exhibited increased salt tolerance of up to 3-fold higher than that of KOD Pol WT (Fig. 5*B–D*). KOD_BR3fingers_ also exhibited greater salt tolerance than KOD Pol WT, but this tolerance was smaller than that of KOD_BR3exo_ and KOD_BR3thumb_ (Fig. 5*E*). These results suggest that the exonuclease and thumb domains and, to a lesser extent, the fingers domains of BR3 Pol contribute to the ability of BR3 Pol to endure under high salt conditions. The inability of any of the chimera KOD proteins to approach the salt tolerance ability of BR3 Pol (∼500 mM NaCl) indicates that all these domains collectively contribute to the salt tolerance mechanism of BR3 Pol. The fact that the intrinsic polymerization activity was intact in the KOD Pol chimera proteins indicates that the introduced charged residues in the chimera KOD proteins might be responsible for their increased salt tolerance ability. Interestingly, the number of unique acidic residues introduced to KOD Pol by swapping the domains from BR3 Pol (18, 15, and 4 for KOD_BR3exo_, KOD_BR3thumb_, and KOD_BR3fingers_, respectively) correlates with the enhanced salt tolerance in the chimera proteins. These results also show that domain swapping to convert nonhalophilic to halophilic polymerase would be an effective engineering strategy among these homologous DNA polymerases.

Testing the activity of these chimera proteins at higher temperatures by PCR amplification yielded important results. Only KOD_BR3fingers_ maintained its activity at higher temperatures, whereas such activity was completely lost in KOD_BR3exo_ and KOD_BR3thumb_ (Fig. 5*F*). These results further support our hypothesis that disulfide bonds in the palm domain are not the primary contributor to thermal stability. They also showed that improving salt tolerance in KOD_BR3exo_ and KOD_BR3thumb_ suppressed their activity at high temperatures. We suspect that this action is caused by the enhanced structural flexibility of the chimera proteins upon introducing the exonuclease or the thumb domains of BR3 Pol.

### BR3 Pol maintains DNA binding at elevated salt concentrations

**Figure 6.**
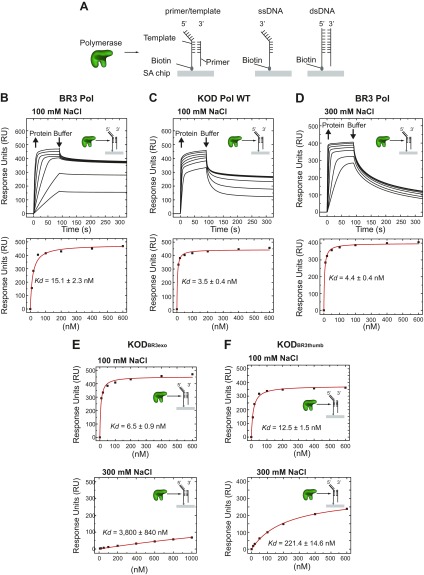
Interactions of BR3 Pol, KOD Pol WT, and KOD chimera proteins with DNA by SPR. *A*) Schematic of the experiment showing the 3 different biotinylated DNA constructs that were immobilized on 3 independent flow cells on a streptavidin (SA)-sensor chip and the injection of the polymerase in solution; a fourth flow cell with no DNA was used to subtract nonspecific interactions between the protein and the surface and the buffer’s refractive index. The 3 tested DNA constructs are primer/template strands (left panel), ssDNA (middle panel), and dsDNA (right panel). *B*) Binding of BR3 Pol to primer/template strands in a buffer containing 100 mM NaCl. Upper panel: serial concentrations of BR3 Pol were injected (10, 20, 50, 100, 200, 400, and 600 nM concentrations) with a surface-regeneration step to remove the bound protein in between. Lower panel: the maximum RUs reached at each protein concentration were fitted using the steady-state affinity mode to obtain the equilibrium dissociation constant (*K_d_*) for each DNA substrate. The uncertainty corresponds to the sd of the fit. *C*) Binding of KOD Pol to primer/template strands in a buffer containing 100 mM NaCl. Experiments were performed and analyzed as in *B* and the protein concentrations used were 10, 20, 50, 100, 200, 400, and 600 nM. *D*) Binding of BR3 Pol to primer/template strands in a buffer containing 300 mM NaCl. Experiments were performed and analyzed as in *B*, and the protein concentrations used were 10, 20, 50, 100, 200, 400, and 600 nM. *E*, *F*) *K_d_* of KOD_BR3exo_ and KOD_BR3thumb_ to primer/template strands in a buffer containing 100 or 300 mM NaCl, respectively. *K_d_* was calculated as described in *B*.

To understand the influence of salt concentration on the DNA-binding affinity and specificity of BR3 Pol, we first characterized the nonspecific interactions of BR3 Pol with ssDNA and dsDNA and the specific interactions with primer/template strands in a side-by-side comparison with KOD Pol, using SPR (**Fig. 6*A***). At 100 mM NaCl, BR3 Pol bound the primer/template strands with a 4.5-fold higher equilibrium dissociation binding constant (*K_d_*) than did KOD Pol (Fig. 6*B, C* and **Table 1**). Both BR3 Pol and KOD Pol discriminated against binding to dsDNA and to a lesser extent against ssDNA. At 300 mM NaCl, BR3 Pol bound the primer/template strands 3-fold more strongly than that at 100 mM and also increased its discrimination against ssDNA to reach that of dsDNA (Fig. 6*B, D*). The faster reduction in RUs of the bound polymerase during the buffer washing step at 300 mM compared with 100 mM suggests that the dissociation rate constant is faster at 300 mM. Therefore, the decrease in *K_d_* at 300 mM is likely due to a significant increase in the association rate constant. The increased specificity to the primer/template structure over ssDNA and dsDNA at higher salt concentrations support our proposition that the decrease in the hydroxyl group interaction from 0 to 0.5 M NaCl might reflect a transition from a dynamic to a more defined and functional structure. KOD Pol WT, conversely, failed to bind the primer/template strands at 300 mM, indicating why it showed no activity at these elevated salt concentrations.

**TABLE 1.** *K*_d_ (nM) of BR3 Pol, KOD Pol WT, KOD_BR3exo_ Pol, and KOD_BR3thumb_ Pol to different DNA structures in a buffer containing 100 mM NaCl or 300 mM NaCl

Heading	BR3 Pol		KOD Pol WT		KODBR3exo Pol		KODBR3thumb Pol	
	100 mM	300 mM	100 mM	300 mM	100 mM	300 mM	100 mM	300 mM
ssDNA	31.6	280.6	32.8	NA				
dsDNA	251.0	178.1	238.6	NA				
Primer/template (nm)	15.1	4.4	3.5	NA	6.5	3800	12.5	221.4

The 3-fold increase in salt tolerance in KOD_BR3exo_ and KOD_BR3thumb_ chimera proteins prompted us to hypothesize that the inserted BR3 exonuclease and thumb domains improved the DNA-binding affinity of KOD Pol. At 100 mM NaCl, KOD_BR3exo_ and KOD_BR3thumb_ bound the primer/template strands but with 2- and 4-fold increased *K_d_* compared with KOD Pol WT (Fig. 6*E, F* and Table 1). At 300 mM NaCl, however, KOD_BR3thumb_ retained binding to the primer/template strands in contrast to no binding by KOD Pol WT. We also observed residual binding of KOD_BR3exo_ at 300 mM NaCl. The weaker intrinsic DNA-binding affinity of KOD_BR3thumb_ and KOD_BR3exo_ at 100 mM suggests that the ability to bind DNA at 300 mM NaCl might result from improved structural adaptation to maintain functional conformation at higher salt concentrations. Collectively, these results indicate that the mechanism of salt tolerance by BR3 Pol could be assisted by its high affinity to the primer/template strands and its ability to retain functional conformation.

## DISCUSSION

Enzyme stability requires structural rigidity, whereas its function requires flexibility to meet required conformational changes during catalysis. It is proposed that enzymes from extreme thermophiles may form rigid structures and rely on temperature variation to balance rigidity with flexibility, whereas enzymes from halophiles may require flexible structures to counteract salt-induced rigid hydrophobic packing. Halophilic proteins would therefore need to balance the structural requirements between being flexible and yet rigid enough to avoid unfolding.

Several mechanisms have been proposed to explain the adaptation of halophilic proteins to hypersaline conditions. Although it is difficult to generalize these mechanisms, it remains widely accepted that increasing the negatively charged residues on the protein’s surface is a common adaptive mechanism (13, 15, 18, 19, 41). Comparison of BR3 Pol with mesophilic yPolD showed an increase in both negatively and positively charged residues of BR3 Pol, with the number of negative residues being significantly higher (Fig. 1*B, C* and Supplemental Fig. 2*B*, *C*). How excess negative charges affect the structural adaptation mechanisms of halophilic proteins is a topic of continued investigation.

Accurate predictions of intrinsic disorder in halophilic archaea species indicate that their proteins are generally rich in disordered regions (63). We predicted the intrinsic disorder of BR3 Pol using RONN and noticed that this polymerase contains significantly more disordered regions than does the mesophilic yPolD (Fig. 2*B* and Supplemental Fig. 6*A*). Secondary structural analysis according to Raman spectroscopy further supported the conclusion that BR3 Pol has more disordered regions than KOD Pol and that these disordered regions mainly consist of random coils (Fig. 2*A*). We used the structure of KOD Pol to make a structural prediction on the location of the disordered regions and negative charges in BR3 Pol. In all, 63 and 36% of the unique Asp and Glu residues, respectively, mapped primarily to the random coils of BR3 Pol (Supplemental Fig. 6*C*). The proposition that Asp increases random coils as an α-helix breaker (51, 52) indicates a correlation between the unique Asp residues and the random coils in BR3 Pol.

**Figure 7.**
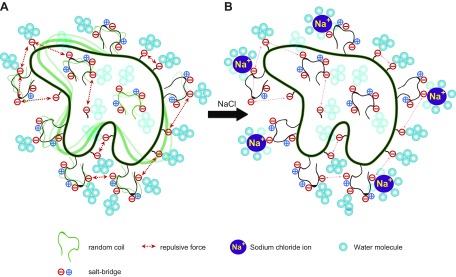
Negatively charged random coils mediate salt-induced transitioning from a flexible to a defined structure. *A*) In the absence of salt, the polymerase experiences dynamic structural fluctuations as a result of its increased percentage of random coils. Excess negative charges (Asp and Glu) on the surface and/or on the random coils mediate this flexibility through repulsive forces. *B*) In the presence of salt, the binding of hydrated Na^+^ ions to acidic residues stabilizes the random coils and reduces the dynamic structural fluctuations leading to the formation of a more functional conformer.

Secondary structure analysis and hydroxyl group interaction as observed by using Raman spectroscopy (Fig. 2A, C) combined with activity and DNA-binding assays (Figs. 3*A* and 6*B, D* and Table 1) at different salt concentrations confirmed that BR3 Pol is structurally flexible. These tests also unraveled how this flexibility mediates the polymerase’s adaptation to hypersaline conditions. Interestingly, salt has a minimal effect on the overall secondary structure of BR3 Pol (Fig. 2*A* and Supplemental Fig. 6*B*), suggesting that the observed higher activity and the specificity of DNA binding upon increasing salt concentration reflects transitioning to a functional structure that is primarily driven by dynamic fluctuations in the protein rather than by disorder-to-order structural transitioning. We propose that repulsive forces among closely located negative charges, in addition to the high percentage of random coils that are negatively charged, provide structural flexibility and dynamism even at elevated salt concentrations (**Fig. 7**). Repulsive forces might also act among different protein molecules to prevent aggregation. This salt-induced structural transitioning might be a universal mechanism that could explain the general trend of salt-induced activation in halophilic proteins (18, 57, 64).

The possibility for extremely thermophilic proteins to adapt lies in the stability of their structures from strong hydrophobic packing, salt bridge interactions, and disulfide bonds (15, 22, 23, 45, 65). Although the overall composition of hydrophobic residues with large or small side chains in KOD Pol and Pfu Pol was not significantly different from that of mesophilic yPolD and even similar to that of BR3 Pol (Supplemental Fig. 2*A*), we found that there was an increase in the percentage of negative and positive charges in KOD Pol, Pfu Pol, and BR3 Pol (Fig. 1*B*). Structural prediction analysis showed that KOD Pol and Pfu Pol exhibited significant reductions in their structural flexibility (Fig. 2*B* and Supplemental Fig. 6*A*), suggesting that they maintained rigid and stable structures. Raman structural analysis on KOD Pol supported these findings by showing significantly fewer random coils (Fig. 2*A*). Although disulfide bonds in the palm domain of extremely thermophilic DNA polymerases have been proposed to play a key role in their thermal stability (38), our findings allowed us to conjecture that this theory is not the case (Supplemental Fig. 3*A*). Our results indicate that polymerases from extreme thermophiles adapt to high temperatures by primarily relying on salt bridge interactions and less flexible structures. Although BR3 Pol is a thermophilic polymerase, it displayed significant structural flexibility compared with KOD Pol. We posit that the primary adaptation mechanism for its thermal stability may be from stabilizing its structure through salt bridge interactions. The balance between structural rigidity for thermal adaptation and flexibility for salt adaptation would be achieved by the relative abundance of salt bridge interactions *vs.* excess negative charges.

Although it has been reported that some DNA polymerases exhibit residual activity in the presence of Zn^2+^ ions (66), BR3 Pol is exceptional in that it can achieve a similar activity level with Zn^2+^ ions as with Mg^2+^ ions but at a significantly lower concentration of Zn^2+^ ions (Fig. 3*E*). Amino acid comparisons showed that the active site contains highly conserved acidic residues that coordinate the metal ions (Supplemental Fig. 1). It is difficult, however, to understand the mechanistic bases of BR3 Pol’s ability to use Zn^2+^ without its crystal structure in complex with DNA. We believe that the higher flexibility of the palm and fingers domains in BR3 Pol might enable it to adapt its conformations in the active site, allowing it to effectively align the incoming nucleotide and the primer/template strands in the presence of Zn^2+^ relative to other DNA polymerases.

The increase in negative charges in halophilic proteins raises a paradoxical puzzle about how halophilic DNA-binding proteins interact with DNA at elevated salt concentrations. Previous studies on the halophilic TBP transcription factor suggest that incorporation of negatively charged residues into the DNA-binding region would sequester cations, allowing the protein to interact indirectly with the DNA phosphate backbone *via* salt bridges (24, 25). This mechanism is appealing because negative charges would also contribute to the adaptation of the protein’s structure to hypersaline conditions. Here, we described an alternative DNA-binding mechanism in which BR3 Pol used the conventional direct DNA-binding mode through a positively charged crevice (Supplemental Fig. 1), which is structurally adapted to maintain functional conformers at elevated salt concentrations.

## Supplementary Material

This article includes supplemental data. Please visit *http://www.fasebj.org* to obtain this information.

## Abbreviations

BR3 Pol: brine pool-3 polymerase
CD: circular dichroism
dNTP: deoxynucleotide triphosphate
dsDNA: double-stranded DNA
KOD: *Thermococcus* *kodakarensis*
ORF: open reading frame
Pfu: *Pyrococcus furiosus*
RU: response unit
SPR: surface plasmon resonance
ssDNA: single-stranded DNA
TBP: TATA box–binding protein
WT: wild type
yPolD: yeast DNA polymerase δ
