# Supplementary material for: Dynamic structure mediates halophilic adaptation of a DNA polymerase from the deep-sea brines of the Red Sea
Source: FASEB J. 2018 Jan 24;32(6):3346–60. doi: 10.1096/fj.201700862RR (PMC6051491; doi:10.1096/fj.201700862RR)
Supplement: Supplementary file 1 [file fj.201700862RR.sf1.pdf]

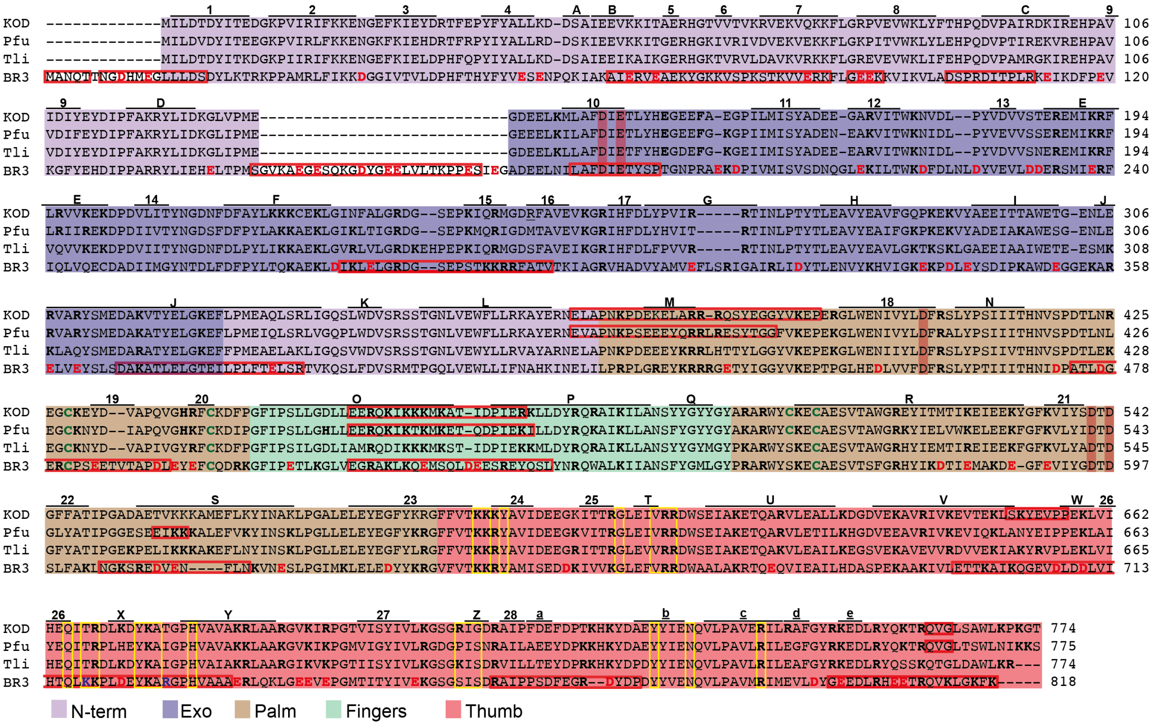

Supplementary Figure 1. Amino acid sequence comparison of BR3 Pol with amino acid sequences of other archaeal polymerases. Multiple sequence alignments of BR3 Pol against KOD Pol (Accession #; 1WNS-A), Pfu Pol (Accession #; WP_011011325.1) and Tli Pol (Accession #; M74198) were performed using ClustalW. The five domains in the polymerase are color coded as indicated. Negatively charged residues that are unique to BR3 Pol are shown in red. Positively charged residues in the exonuclease, palm, fingers and thumb domains are shown in bold. Cysteine residues in the palm domain expected to be involved in disulfide bonds are shown in green. Disordered regions predicted by RONN are outlined in red (see Fig. 2*B* and Supplementary Fig. 6*A*). Residues that are in direct contact with the DNA in the thumb domain are boxed in yellow. They are 20 residues within 3.6 Å of the DNA. Almost all these residues are conserved in BR3 Pol, except for Thr667 and Arg709 in KOD Pol, which are, respectively, replaced by Lys718 and Ser760 in BR3 Pol.

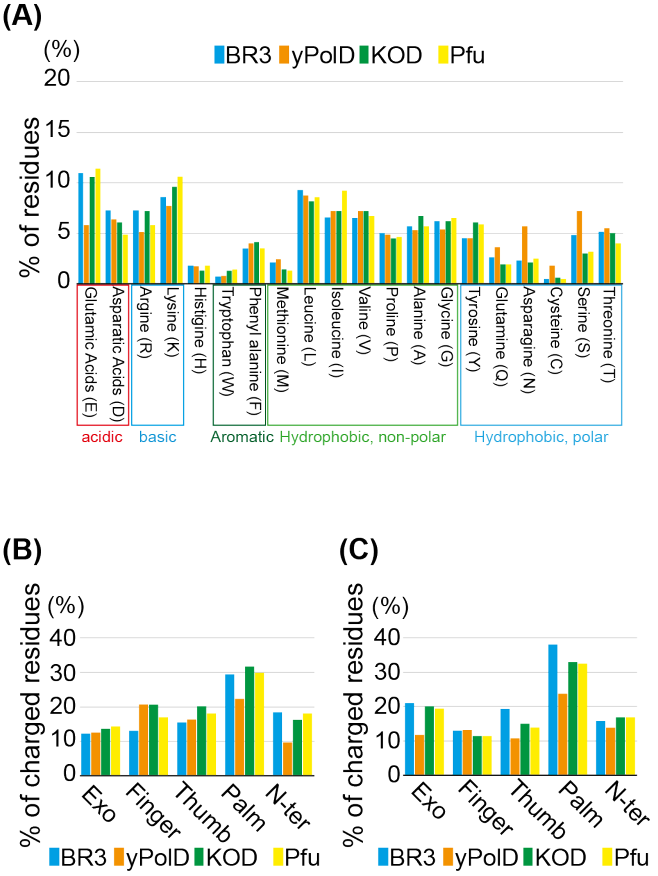

Supplementary Figure 2. (A) Percentage of each amino acid in the overall protein sequence. The number of each amino acid residue is counted and percentages are shown for BR3 Pol, yPolD, KOD Pol and Pfu Pol. (B), (C) Charged residue traits of BR3 Pol, yPolD, KOD Pol, and Pfu Pol. The number of positively charged residues (basic residues: Arg and Lys) (B**)** and negatively charged residues (acidic residues: Glu and Asp) (C**)** are counted and percentages are shown for each of the five domains in the polymerase.

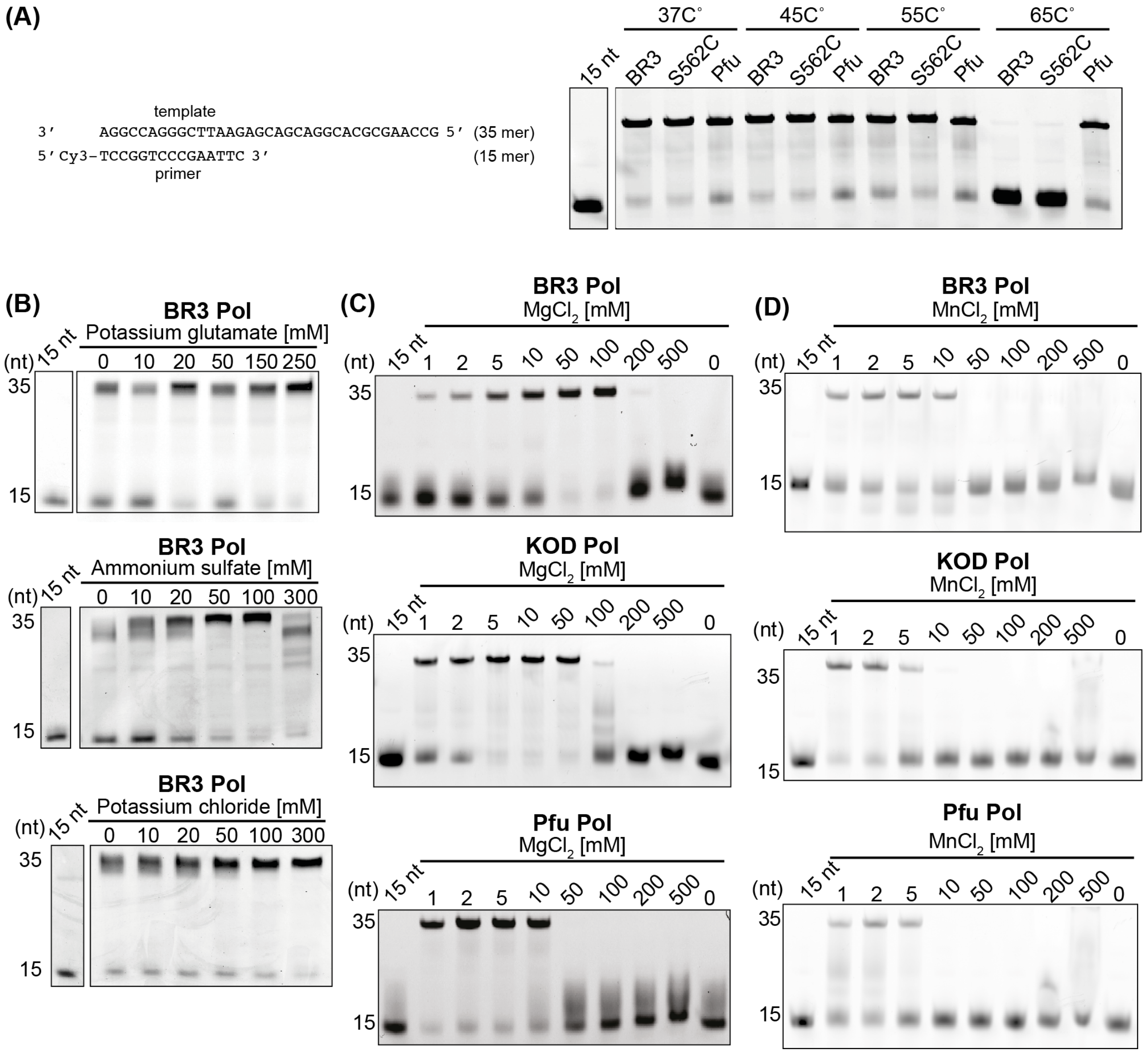

Supplementary Figure 3. (A) Temperature-dependent polymerization activities of BR3 Pol, BR3(S562C) Pol and Pfu Pol. Polymerase activity was measured on a short primer/template substrate consisting of a 15-mer primer labeled at its 5’ end with Cy3 and a 35-mer template strand (inset). Reactions were performed, as described in MATERIALS AND METHODS section, in a buffer containing the optimal concentration of NaCl. Products were analyzed on a denaturing 20% polyacrylamide urea gel. Polymerases were pre-incubated at the indicated temperature for 10 min and its polymerization activity was measured as described in Fig. 3*A*. BR3(S562C): Serine at position 562 in BR3 Pol was replaced with Cysteine to introduce an extra disulfide bond. (B) The salt-dependent polymerization activity of BR3 Pol in the presence of potassium glutamate (upper panel), ammonium sulfate (middle panel) and potassium chloride (lower panel). (C) Effect of increasing $MgCl_2$ concentration on the polymerization activities of BR3 Pol (upper panel), KOD Pol (middle panel), and Pfu Pol (lower panel). (D) Effect of increasing $MnCl_2$ concentration on the polymerization activities of BR3 Pol (upper panel), KOD Pol (middle panel), and Pfu Pol (lower panel). Reactions (C and D) were performed and analyzed as in Fig. 3*A* in a buffer containing no salt. These assays were conducted under optimal salt concentrations for each polymerase.

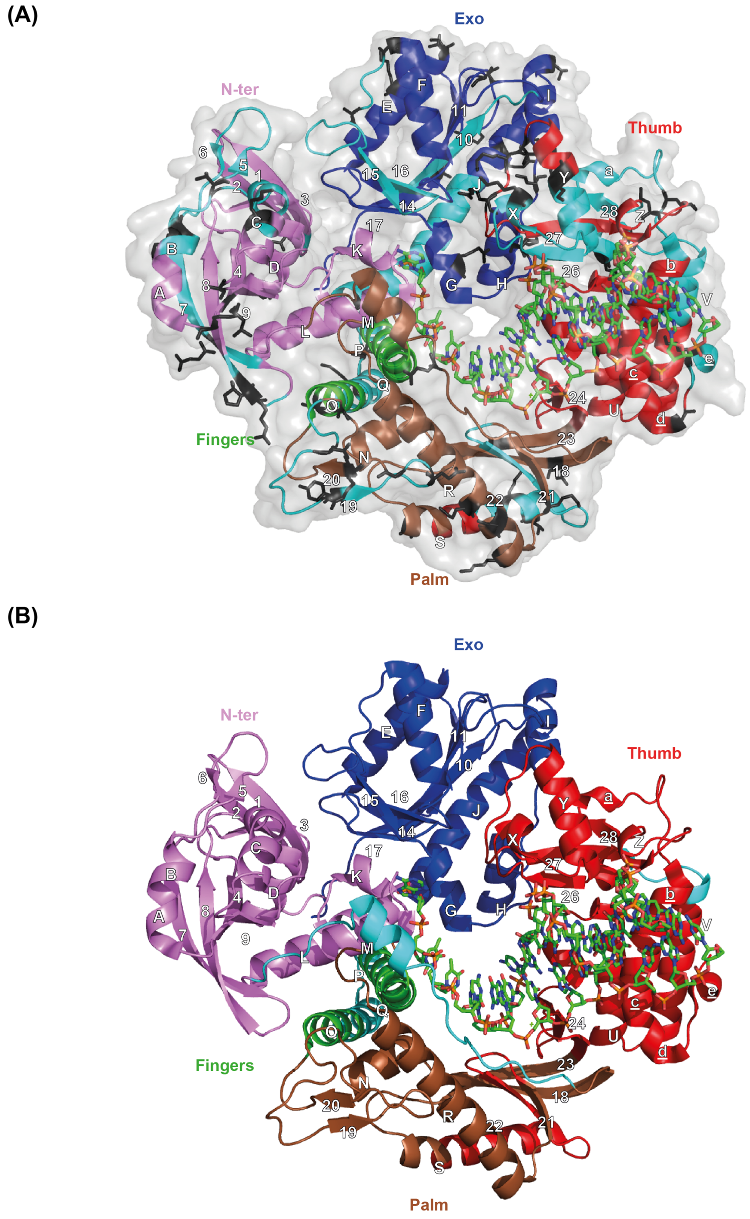

Supplementary Figure 4 Disordered regions and unique acidic residues in BR3 Pol are primarily exposed to its surface. (A) Predicted locations of disordered regions (cyan) and unique acidic residues (black sticks) of BR3 Pol in the crystal structure of KOD Pol (4K8Z). The protein and DNA are depicted as described in Fig. 1*A*. Amino acid alignment between BR3 and KOD Pol was performed by using ClastalW (see figure legend in Supplementary Fig. 1), and the disordered regions and unique acidic residues in BR3 Pol are mapped to the corresponding locations on the crystal structure of KOD Pol. The residues of KOD Pol that were replaced with unique acidic residues (Glu or Asp) in BR3 Pol are shown as black sticks. The residues represented by black sticks are mainly exposed to protein surface. The molecular surface of KOD Pol is indicated by transparent gray. The disordered regions of BR3 Pol predicted by RONN are shown in cyan (see Fig. 2*B* and Supplementary Fig. 6*A*). (B) Locations of disordered regions in the crystal structure of KOD Pol (4K8Z). The protein and DNA are depicted as described in Fig. 1*A*. The disordered regions of KOD Pol predicted by RONN are shown in cyan (see Fig. 2*B* and Supplementary Fig. 6*A*).

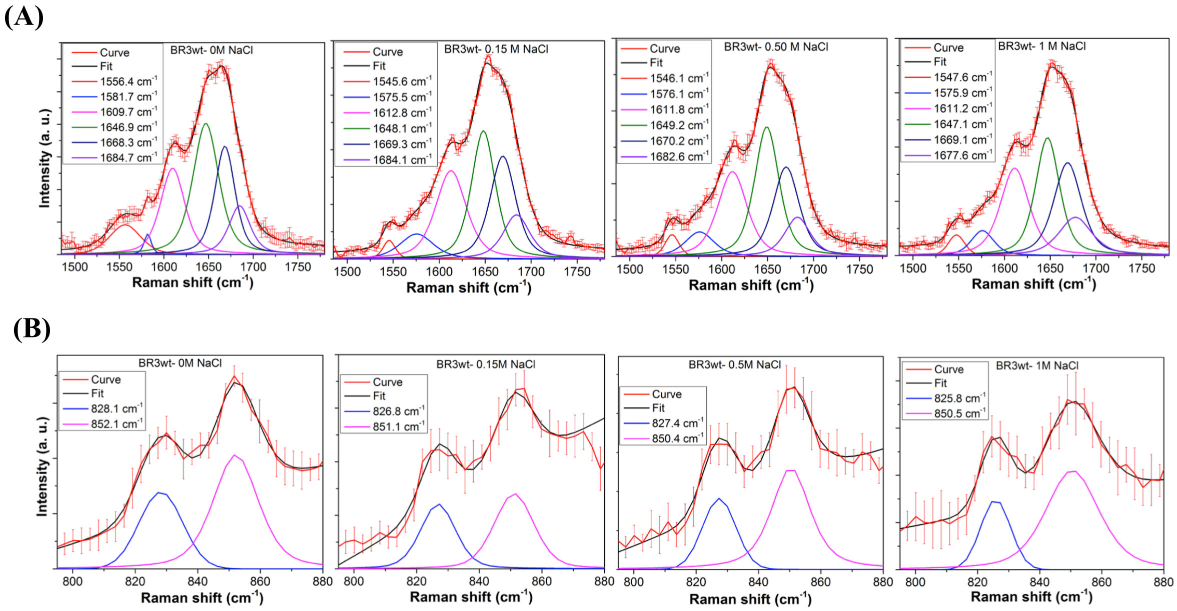

Supplementary Figure 5. (A) Curve fitting for BR3 Pol with varying NaCl concentration (0 M, 0.15 M, 0.5 M and 1.0 M) in the range of 1500 – 1800 cm^{-1}. The curve fitting was performed by choosing six curves of mixed Gaussian and Lorentzian distribution functions. Secondary structures (α-helix, random coil, β-sheet) of BR3 were estimated by calculating the integrated areas of the peaks centred at around 1650, 1665 and 1680 cm^{-1}, respectively. (B) Curve fitting for BR3 Pol with varying NaCl concentration (0 M, 0.15 M, 0.5 M and 1.0 M) in the range of 800 – 880 cm^{-1}. The behaviour of Tyr phenolic hydroxyl group interaction with hydrogen bonds in BR3 Pol was estimated after curve fitting of doublet of Tyr bands centred at 830 and 850 cm^{-1}. Two curves of mixed Gaussian and Lorentzian distribution functions were used to carry out the curve fitting.

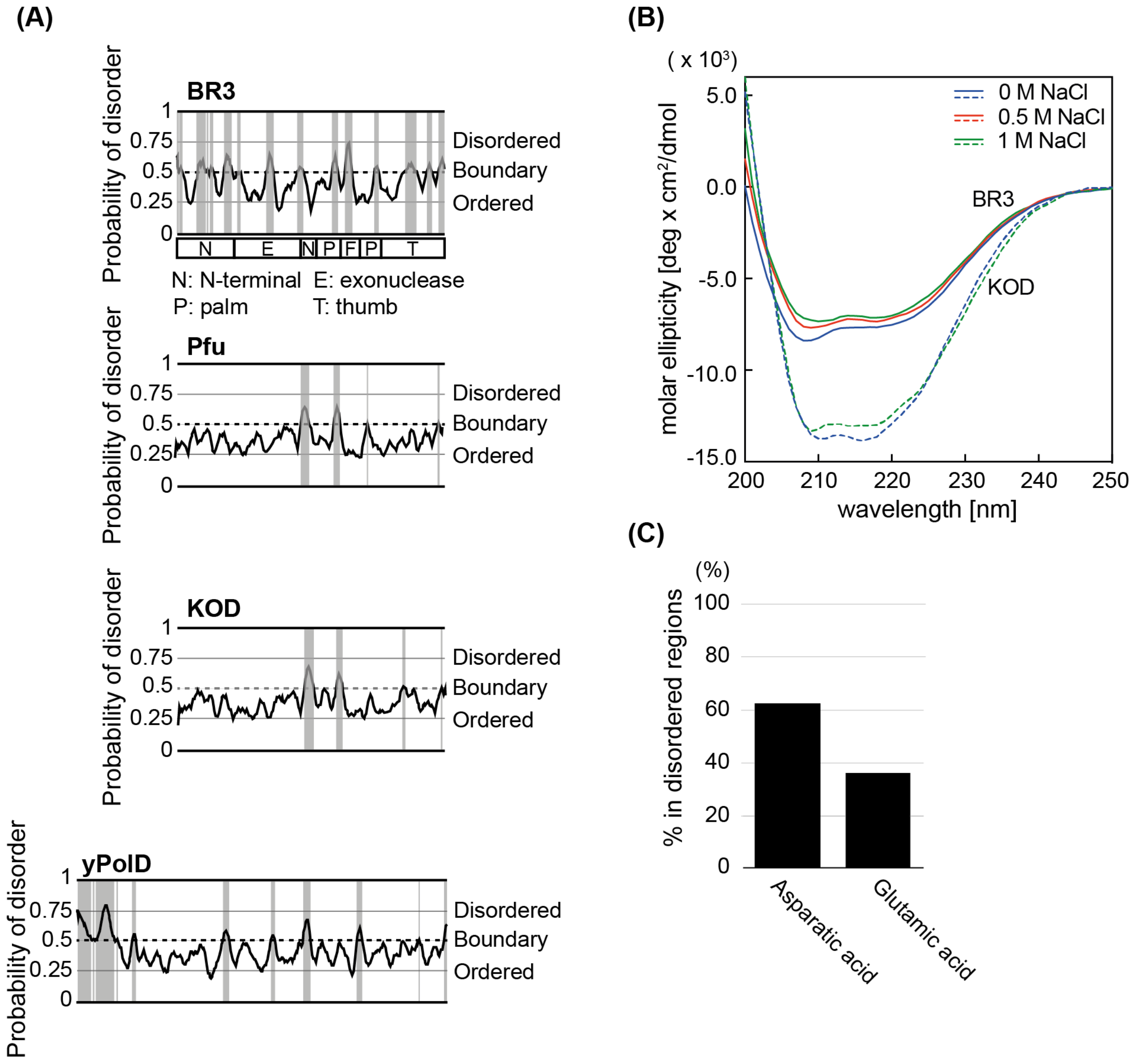

Supplementary Figure 6. (A) Plot of disorder probability calculated by RONN for BR3 Pol, Pfu Pol, KOD Pol, and yPolD. The horizontal dashed lines mark the threshold for disorder prediction and the disordered regions are shown in shaded grey boxes. (B) Far-UV CD spectra as a function of salt. CD spectrum of BR3 Pol (solid lines) and KOD Pol (dashed lines) in PBS are shown. NaCl concentration was 0 M (blue line), 0.5 M (red line), and 1M (green line). Measurements were carried out at a wavelength of 200 to 250 nm at 20 ℃. (C) Percentage of the unique Asp and Glu residues in the disordered regions of BR3 Pol.

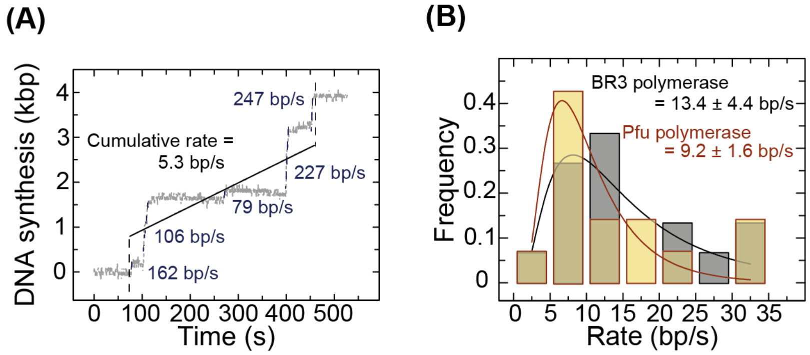

Supplementary Figure 7. Cumulative rates of DNA synthesis by BR3 Pol and Pfu Pol in single-molecule primer extension reactions. (A) A representative trace of primer extension reaction by Pfu Pol. The accumulative rate of DNA synthesis was calculated by fitting the entire trajectories including the pause duration. (B) Frequency distribution of cumulative rates of DNA synthesis by BR3 Pol and Pfu Pol fitted with a Gaussian distribution (13.4 ± 4.4 b/s (N = 15), 9.2 ± 1.6 b/sec (N = 14), respectively). The rates were calculated using the same single-molecule trajectories used in Fig. 4.

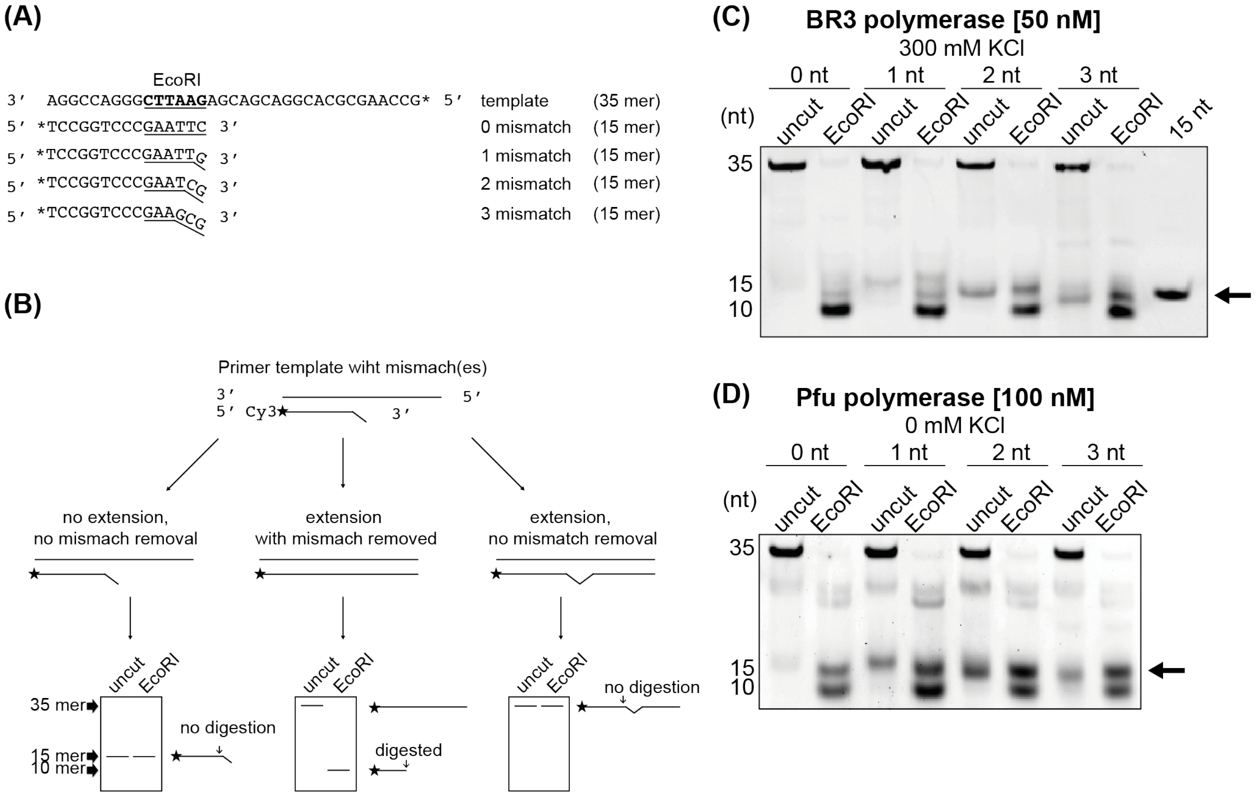

Supplementary Figure 8. Exonuclease proofreading activity assay. (A) DNA sequences of the 35-mer template strand and the 15-mer primers with 0, 1, 2 or 3 mismatched nucleotides located at the EcoRI site are shown. (B) Diagram explaining the various possibilities of how the polymerase deals with the primer/template substrate in absence or presence of a mismatched nucleotide and the resulting bands that would be detected on the denaturing polyacrylamide gel upon product digestion with EcoRI. The presence of a fully extended product (35-mer) in the uncut lanes reflects extension of the mismatched primers. The EcoRI digested band (10-mer) of the extended products show that the EcoRI recognition site is restored due to the removal of 3' mismatched nucleotide(s) on the primer. The unextended 15-mer primers are from substrates where the polymerase failed to remove the mismatch. (C) Proofreading activity by BR3 Pol; 50 nM BR3 Pol was used and reaction was performed in buffer containing 300 mM KCl. The unextended 15-mer primers are shown by arrow. (D) Proofreading activity by Pfu Pol; 100 nM Pfu Pol was used and reaction was performed in a buffer containing 0 mM KCl. The unextended 15-mer primers are indicated with arrows. A two-fold higher concentration of Pfu Pol was required to achieve the same proofreading activity of BR3 Pol. Proofreading assays were conducted as described in the MATERIALS AND METHODS section.
